# Chemical Composition of Areca Nut and Its Adverse Effects on Human Health

**DOI:** 10.7759/cureus.43739

**Published:** 2023-08-19

**Authors:** Suwarna Dangore-Khasbage, Rahul R Bhowate, Monika Khubchandani

**Affiliations:** 1 Oral Medicine and Radiology, Sharad Pawar Dental College and Hospital, Datta Meghe Institute of Higher Education and Research, Wardha, IND; 2 Pediatric Dentistry, Sharad Pawar Dental College and Hospital, Datta Meghe Institute of Higher Education and Research, Wardha, IND

**Keywords:** policy, harmful, toxicity, components, oral cancer, adverse effects, oral submucous fibrosis, copper, alkaloids, areca nut

## Abstract

Areca nut (AN) is one of the addictive substances consumed widely in the world. The composition of AN is very complex, and each component has variable properties. This study aims to review the composition of AN and its adverse effects on humans. For this review, the literature search was performed by an electronic search of the Pubmed/Medline, Scopus, and Google Scholar databases using proper MESH headings and retrieved the articles published from 1997 to 2022. The eligibility criteria included human studies, a form of AN, the composition of AN, harmful effects of AN, the effect of AN on the oral cavity, the effect of AN on vital organs, and articles published in English. Data were extracted regarding the composition of AN, forms of AN, and harmful effects of AN on the oral cavity and on other systems. A total of 449 articles were identified from various databases, and 36 studies were selected that met the inclusion criteria. The active components of AN, which produce harmful effects, are primarily alkaloids, polyphenols, tannins, and certain trace elements. AN is consumed in different forms, and based on the form, its composition also varies. AN is known to cause deleterious effects on the oral cavity as well as various body organs. The most dangerous and widely reported impacts of AN on the oral cavity are the development of oral submucous fibrosis, a premalignant condition, and oral malignancy. However, during the chewing process, excretory products of AN are released and circulate in the body of "chronic habitual" and affect the other body parts. Thus, AN consumption may contribute to cardiac, liver, endocrinal, metabolic, respiratory, and reproductive system disorders also. AN composition is complex, and its consumption is harmful to human health. In regard to controlling the issue of the harmful effects of this habit, preventive measures should be established.

## Introduction and background

Areca nut (AN) is a seed of the areca catechu palm tree, cultivated in most of the tropical Pacific, Asia, and parts of East Africa. Chewing AN is an old tradition that dates back a thousand years and is associated with people's culture [1]. References to AN chewing habit are available in ancient Greek, Sanskrit language, and Chinese literature. In Sanskrit, the manuscript described the usage of AN in food, medicine, and social or religious ceremonies [1,2].

AN is a popular substance resulting in addiction after chronic use. Its origin is primarily reported in South East Asia and India and then spread to countries all over the world due to the migration of these populations. Now AN consumption has become a familiar habit in many Asian countries and migrated communities in Africa, Europe, the UK, North America, and Australia [3,4]. Approximately 10% of the population of the world consumes AN regularly. AN ranks fourth position among the widely used psychoactive substances [5,6]. Chewing AN evokes euphoria, a feeling of happiness that contributes chiefly to the addiction and popularity of this custom.

AN is consumed at different stages of its maturity, in various forms, and in a variety of combinations, such as with or without tobacco. Irrespective of the maturity stage, form, or combination, AN is reported to be harmful to human beings. The harmful manifestations are seen in the oral cavity and various vital organs. In India, AN consumption is a matter of worry as 20-30% of the population is AN habitual [3,5]. The increasing prevalence of AN habit among youngsters is highly noticed. An initiative to curb the usage of AN among people by making them aware of its adverse effects is the prime accountability of oral health care providers. The present article describes the toxic effects of AN with descriptive explanations for the same.

The present article describes the chemical composition of AN in detail. The role of all the components of the AN that are responsible for its toxic effect along with the mechanisms relevant to toxicity due to AN consumption is elaborated.

## Review

Search methodology (data source and search strategy)

The first step of the search strategy comprised an electronic search of PubMed/Medline, Scopus, and Google Scholar databases of the National Library of Medicine, National Institutes of Health, Bethesda, Maryland. Databases were searched from 1997 up to and including 2022 using various combinations of the following keywords: “AN,” “betel nut,” “areca catechu,” “AN habitual,” “betel nut habitual,” “betel nut chewing,” “oral submucous fibrosis (OSMF),” “oral cancer,” etc. The second step was to hand‑search the reference lists of original and review articles that were found to be relevant in the first step. Titles and abstracts of articles obtained using the above‑described search protocol were screened by each author. Only full-text articles were selected after assessing their eligibility and checking for agreement. Any disagreements between the authors were resolved via discussion. All the well‑designed original studies published in English that covered the effect of AN consumption on the oral cavity and vital organs were selected. The search strategy used in PubMed was as under. ([[[[areca* [Title/Abstract]] odds ratio [OR] catechu* [Title/ Abstract])] OR “betel nut*” [Title/Abstract]] OR “Areca” [Mesh]) AND ([[[[[[[[[[[[[“AN habitual”[Title/Abstract]] OR “betel nut habitual”[Title/Abstract]] OR “betel nut chewing” [Title/Abstract]] OR "Oral Submucous fibrosis” [Title/Abstract] OR "Oral fibrosis” [Title/Abstract] OR "Oral malignancy” [Title/Abstract] “oral cancer” [Mesh]).

Inclusion and Exclusion Criteria

With the consensus of all the authors, the following eligibility criteria were decided for the selection of articles: (1) human studies, (2) types and form of AN, (3) composition of AN, (4) the effect of AN on the oral cavity, (5) the effect of AN on vital organs, and (6) articles published in English. All prospective, retrospective, case-control, community‑based, and hospital‑based studies were included in which adverse effects of AN consumption were assessed. Studies on animal models, unpublished articles, letters to the editor, review articles, case reports, and books were excluded.

Result

**Figure 1:**
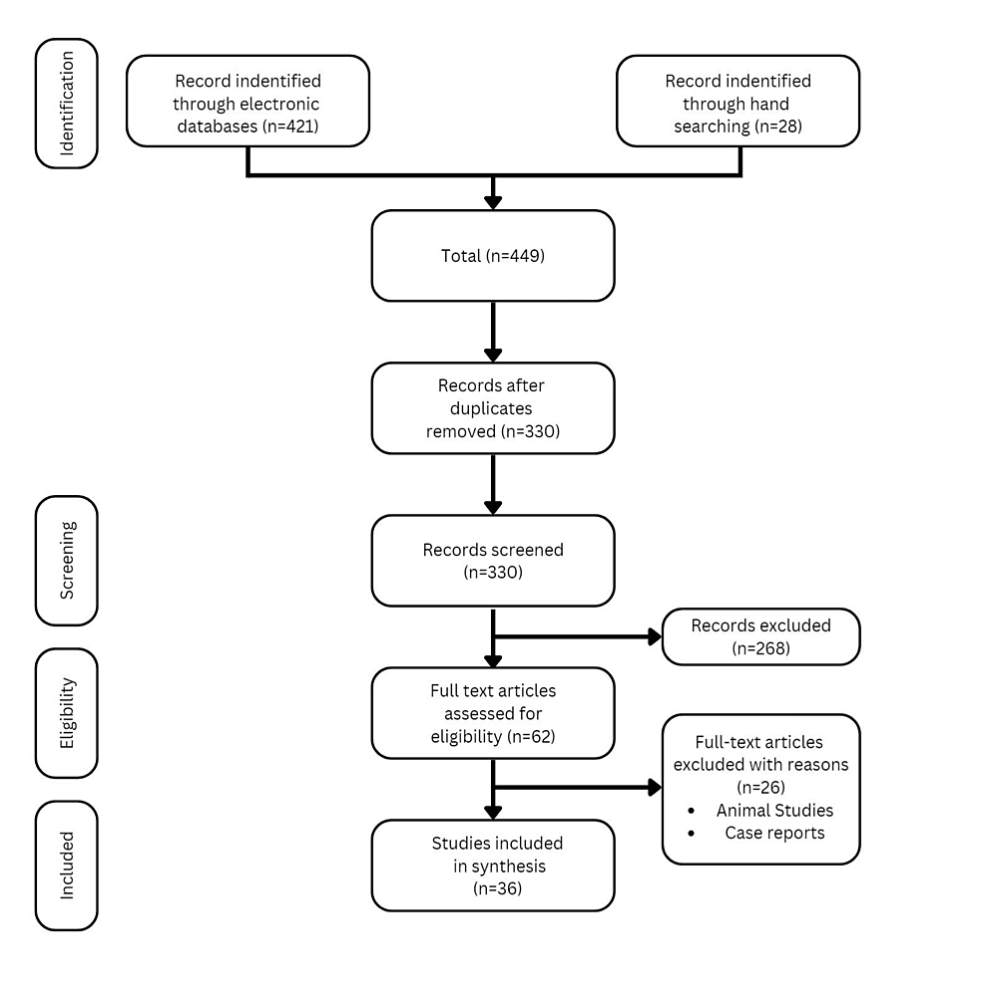
Showing the "Preferred reporting items for systematic reviews and meta-analyses" flowchart

In total, 449 articles were retrieved from electronic databases (PubMed/Medline: 118, SCOPUS: 96, Google Scholar: 130, EMBASE: 67, and manual searching: 38). Out of these, 330 titles remained for screening after the removal of duplicates. On review of the titles and abstracts of all these articles, 268 publications were excluded because these did not report the harmful effects of AN. Consequently, 62 full‑text articles were then reviewed to assess their eligibility, out of which, 26 were excluded because these were case reports or articles with animal studies. Ultimately, 36 articles that met the eligibility criteria were included in the review. Figure 1 depicts the "Preferred reporting items for systematic reviews and meta‑analyses" flowchart showing a flow of information.

AN Production as Well as Consumption in India

India ranks first concerning the area and production of AN. Approximately half of global AN production also takes place in India [7]. The maximum AN producer state is Karnataka accounting for 62.69%, followed by Kerala and Assam. The livelihood of approximately 10 million Indians is dependent on the AN industry [2]. Another significant factor is that AN is one of the substantial export businesses in India. There are plenty number of AN industries in India. Overall, the AN industry and AN business influence low socio-economic people, as they derive their income from this business. Thus, eradicating the AN trade is a matter of concern in India. Similarly, the Indian scenario concerning the habit of AN consumption is more worrisome, as consumption is reported to be nearly 20-30% of the population in the last two to three decades.

According to the National Family Health Survey, both males and females consume AN, either with tobacco or without tobacco in an age range of 15-49 years. In India, AN is usually chewed with tobacco in many states [7]. Rather, AN consumption is a part of typical societal customs. It is also consumed for its beneficial effects such as improvisation of concentration, stress reliever, mouth freshener, and digestive agent [5,8].

Epidemiological surveys of the past two to three decades showed the prevalence of betel quid (BQ) consumption in India, Nepal, and Pakistan as 20-40% [9]. Nevertheless, profound advertisement, nearby availability, and low price of commercially manufactured forms of AN have made it very approachable to youth, children, and teenagers in India [10]. Its consumption is so prevalent in India that it might be the second most commonly consumed carcinogen in the Indian subcontinent [8].

The first Global Adult Tobacco Survey for India (GATS 1-2010) showed that AN with tobacco is consumed by men and female both, with a more prevalence of men. The mixtures of AN and tobacco, without betel leaf, were used by more men than women. In rural areas, BQ with tobacco, gutka, and similar product consumption was higher than in urban areas. Region-wise prevalence of BQ with tobacco was more in the northeast and east, while in the north, it was lowest. On the other hand, the popularity of gutka and preparations like this was greater in the central states (12.1%) [11].

The second “Global Adult Tobacco Survey” for India (2016-17) reported that the prevalence of tobacco use is reduced by 6% from GATS 1 to GATS 2. It is reported that 28.6% of adults above 15 years of age consume tobacco might be in any form. Among men, the most commonly consumed products are khaini, bidi, and gutka, while women chew BQ with tobacco. The expenditure on cigarettes has tripled, and that on bidi and smokeless tobacco has doubled since GATS 1. Specifically, 55% of smokers and 50% of smokeless tobacco users are planning to quit the habit [12]. GATS 2 reports a reduction in the prevalence of the use of tobacco in the young population.

Different Forms of AN Consumption

AN may be consumed at its different stages of ripening. It is used when it is green (unripe stage), like a small olive, and without tobacco in Taiwan [13-15]. In India, AN is used in dry form, commonly called "supari." The areca fruits are dried in sunlight for numerous weeks, and then their fibrous shell is removed and then used for consumption. As AN is hard to consume, it is cut into fine pieces and then consumed. Sometimes, ANs are fermented before removing the shell by keeping them in moist pits for a few weeks and then used for consumption. This variety of AN is called "kwai" or "tambul" widely used north-eastern part of India. As it is relatively soft, larger pieces of the nut are consumed by the chewer [13,16]. In India, processed AN is available as "red supari" and "white supari." Both forms are obtained by boiling and drying de-husked AN. In the former type, unripe AN is used, while in the latter type, ripe AN is used. When AN is used with other ingredients, the preparations are named BQ, mawa, gutka, and pan masala [4]. One of the AN preparations used in India is called "sweet or scented supari." This contains AN, artificial sweeteners, some spices, and mint as a flavoring agent and coloring agent.

In India, AN is preferably consumed along with tobacco, and the preparation is named BQ [4,16]. AN is wrapped in the Piper betel plant's leaf. Thus, the common name is betel nut. However, "betel quid" or "betel nut" is the wrong terminology frequently used for AN. The betel tree does not contain fruits but contains only leaves, called betel leaves [17]. Nevertheless, AN is a fruit of a palm tree named "areca catechu." In other specific areas such as Pakistan, Bangladesh, and Sri Lanka, also the AN is consumed with slaked lime, flavorings, and cut tobacco leaves that are wrapped in betel leaf [13,16]. Apart from this, AN is available as a pan masala. Pan masala is an industrially manufactured AN-containing product. Pan masala containing tobacco is referred to as gutka [4]. Whatever may be the form, AN consumption is unsafe for oral and general health.

AN habit is stated to be a gateway for tobacco habit because, in children, chewing habit usually begins with chewing sweetened and flavored AN products. This, later on, progresses to tobacco chewing [18]. It is reported that, among school-going children, AN users are multiple times more likely to be tobacco users as compared to non-users of AN. Indeed, aggressive advertising, effortless availability, and low price of its preparation attract people, including children and teenagers in particular. One of the widely consumed, an industrially manufactured AN product with tobacco named "gutka," is banned in India in almost all the states and union territories. Still, it is accessible to the population with no effort [9].

Constituents of AN

**Figure 2:**
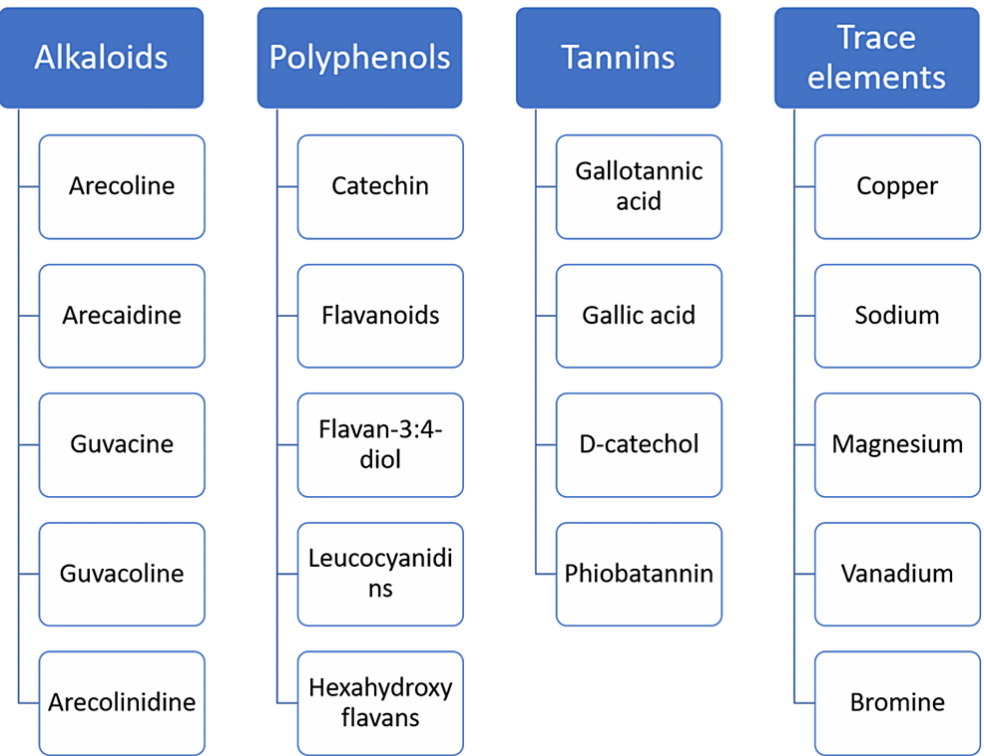
Showing the chemical composition of areca nuts

The contents of AN are very complex, and each component has variable properties. The active components of AN, which produce harmful effects, are primarily alkaloids, polyphenols, tannins, and certain trace elements [10], as shown in Figure 2.

AN contains rudimentary fibers, carbohydrates, fats, alkaloids, polyphenols, tannins, proteins, and water. The active components of AN, which produce harmful effects, are primarily alkaloids, polyphenols, tannins, and certain trace elements [10].

Even though several chemicals are present in AN, with relevance to biological actions, arecoline and arecadine are important. Arecoline is in plentiful quantity than arecaidine. Polyphenols constitute mainly the dry weight of the nut. The content of AN may vary based on its maturity as well as the processing method. For example, tannin is maximum in unripe AN and minimum in matured AN. Likewise, the maximum tannin content is reported in a roasted nut, while the minimum in boiled nuts [2].

One of the harmful trace elements present in AN is copper (Cu). It is stated that the total intake of Cu may exceed the actual requirement in chronic AN chewers. Especially in commercially available processed AN, the Cu concentration is higher than the raw AN. The cellular metabolism of AN generates reactive oxygen species (ROS) at a pH of more than 9.5, which is harmful to the human body [10,19].

Harmful Effects of AN on Oral and General Health

AN is reported to have many hazardous effects on the human body in general, as well as on the oral cavity [3,5,20,21]. The most dangerous impacts of AN on the oral cavity are the development of OSMF, a premalignant condition, and oral malignancy [3,17,20]. “World Health Organization” and “International Agency for Research on Cancer” classified AN as a “Group 1 human carcinogen,” providing adequate evidence stating its role in OSMF, oral, pharyngeal, and oesophageal malignancies [8]. 

Based on the available literature, multiple reasons are there that explain the role of AN in the causation of these conditions. Few of these are attributable to the presence of alkaloids, polyphenols, Cu, and aflatoxin in AN. Among alkaloids, both arecoline and arecadine are responsible for fibroblast proliferation and increased collagen formation, one of the pathogeneses reported in OSMF. Polyphenols of AN, such as flavonoids, catechin, and tannins, result in excessive fibrosis with an insoluble, stable collagen formation as it inhibits collagenase activity. This prevents collagen breakdown, which, in turn, adds to increased fibrosis. This is the basic pathogenesis of OSMF [10,20]. The flavonoid content of the AN enhances collagen production, toughens cross-linking, and diminishes the degradation of collagen by various molecular pathways, as reported in OSMF. Similar mechanisms take place in the liver as a result of the circulation of excretory products of AN after chewing. Accordingly, the presence of alkaloids in the blood is reported in the previous study [22].

**Figure 3:**
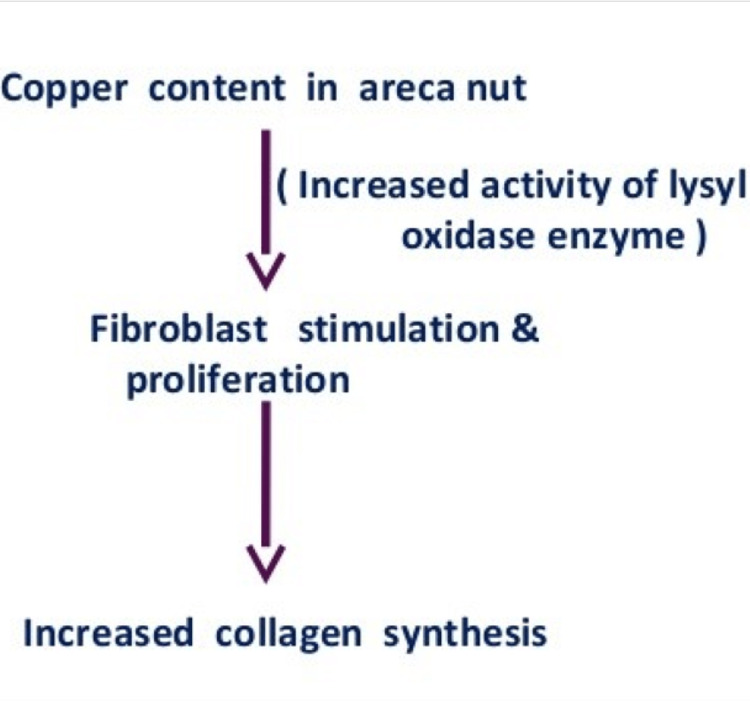
Mechanism of copper content of areca nuts in oral submucous fibrosis

A significant amount of Cu present in AN takes part in the pathogenesis of fibrotic conditions. It enhances the functioning of lysyl oxidase, a Cu-dependent enzyme. Cu upregulates fibroblastic activity in the form of collagen synthesis, collagen cross-linking, and inhibition of collagen degradation [23], as shown in Figure 3.

It is stated that approximately 5 mg of Cu is consumed every day by an adult Indian who consumes AN on a daily basis. If the consumer has the habit of swallowing the quid juice, then a considerable amount of Cu is swallowed [23]. This excess Cu is harmful as it induces several fibrotic conditions, including oral submucous fibrosis [23]. Nonetheless, high serum Cu levels are reported in AN chewer suggesting its role in systemic conditions such as liver fibrosis. Additionally, the high Cu content generates ROS that triggers hepatocellular-damaging effects, which become significant when the capacity of the liver to maintain these metals in storage forms is exceeded.

In addition to the above, a few other harmful chemicals such as polycyclic aromatic hydrocarbons, calcium hydroxide, and nicotine may be introduced in AN during its processing or to its various preparations, such as BQ, gutka, and pan masala [9]. Besides this, during the chewing process, certain harmful chemicals are produced due to the reaction between areca alkaloids with salivary nitrite [9].

As per the earlier estimation by the “International Agency for Research on Cancer” (1985), consumption of BQ in combination with tobacco was reported to be harmful to humans as a carcinogen. However, the assessment of 2004 concluded that chewing AN alone is also carcinogenic to humans [4,13,14]. A basic idea about the etiological role of AN in oral cancer is emerged from Taiwan, as most of their preparations are tobacco-free. Nearly 10% of people consume AN only, which is without tobacco. Still, cases of oral cancer are reported in Taiwan [14]. Commercial man-made products such as gutka and pan masala are more hazardous [3,4].

Up to the 1980s, data reported that OSMF is a rare disorder affecting older AN chewers. However, in 1990, the situation totally changed, and OSMF was reported to be a common disease of the younger population This sudden increase in OSMF was due to the introduction of industrially prepared and marketed AN and tobacco preparations. Even for advertisements, electronic media were explicitly used to target and attract the youth [24].

OSMF is a chronic oral mucosal disease prevalent in India and Taiwan and occasionally affects Europeans. It basically affects the oral mucosa, followed by pharyngeal mucosa and esophagus. Chronic inflammatory reaction reduces fibro-elasticity of oral mucosa and then the occurrence of epithelial atrophy. The burning of the oral mucosa and the inability to eat hot and spicy food are the complaints of the patient in the initial stage of the disease. Formation of fibrous band results in the inability to open the mouth completely. This altogether hampers food intake, maintenance of oral health, and ability to speak. Fibrous bands may affect any surface of the oral cavity, including buccal mucosa, retromolar areas, soft palate, and oropharynx. Instead, the presence of fibrous bands is one of the diagnostic criteria for OSMF, and the involvement of the number of oral mucosal surfaces directly correlates with the severity of the disease. A smooth-appearing tongue characterizes the tongue's involvement due to the absence of papillae and restricted movements. Depending on the presence of fibrous bands and degree of mouth opening, Haider et al. [25] have reported clinical and functional staging to be used in OSMF. The typical facial appearance and presence of certain systemic features of OSMF are altogether termed gutkha syndrome by Chaturvedi [26].

**Figure 4:**
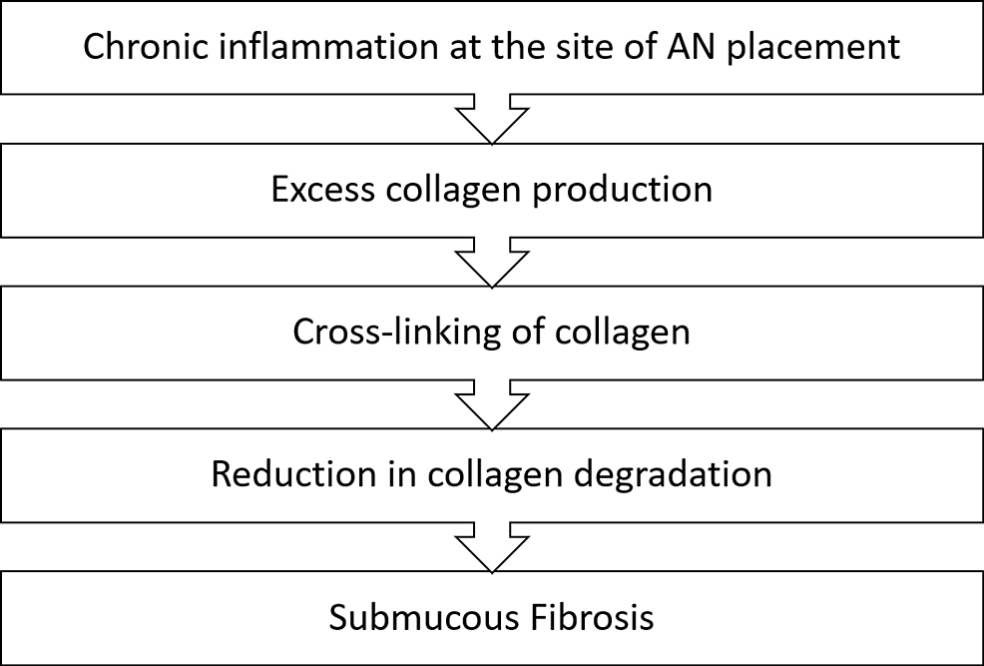
Stepwise pathogenesis of oral submucous fibrosis

The etiology of OSMF is multi-factorial, but AN consumption is the primary and well-accepted causative factor reported in the literature. Figure 4 describes the stepwise pathogenesis of OSMF [20].

The first step in the pathogenesis of OSMF is oral mucosal irritation both chemically and mechanically. Chemically irritation is by alkaloids and flavonoids present in AN, which are absorbed and undergo metabolism. Mechanical irritation is because of the crude fibers in AN. Persistent inflammation is the critical step in the pathogenesis of OSMF and oral cancer.

The second step involves the activation of pro-collagen genes and an increase in pro-collagen proteinase enzyme, resulting in increased collagen synthesis.

The third step is collagen cross-linking and “insoluble collagen formation” with the help of lysyl oxidase and flavonoid. Lysyl oxidase is a Cu-dependent enzyme, and as AN contains Cu, AN consumption facilitates the functioning of lysyl oxidase. Flavonoids in AN also play an essential role in enhancing the cross-linking of the fibers. Thus, Cu and flavonoids in AN increased cross-linking of the collagen fibers by enhancing lysyl oxidase activity.

In the fourth step, tissue growth factor β hampers collagen degradation by activating the “tissue inhibitor of matrix metalloproteinase gene” and “plasminogen activator inhibitor gene.” Overall, sequela is submucosal fibrosis.

Another more serious AN-induced condition is oral cancer. It is said that, in India, the prevalence of oral cancer is four in 10 of all cancers. The reason for this is the popularity of addictive habits due to cultural, ethnic, and geographic factors among Indians. Annually 130,000 deaths are reported to be due to oral cancer in India. Recent data indicate the prevalence of oral cancer in the young population [2,5].

The pathogenesis of oral cancer is also related to the chemical composition of AN, such as alkaloids and polyphenols. Polyphenols are potential carcinogens. The interaction between AN-containing carcinogens and cellular molecules is a key event in chemical carcinogenesis. However, these are not the only factors. The production of ROS in the saliva of AN chewers also has a significant role in oral carcinogenesis. Nitrosamines that are specific to AN are demonstrated to be tumorigenic, as these can cause genetic changes in the cells [27]. Hu et al. [28] demonstrated the significance of direct-acting alkylating agents in AN, especially commercial AN products. These agents are present in AN at a level that is likely to cause DNA damage.

These harmful effects of AN are not constrained to the oral cavity only. However, during the chewing process, excretory products of AN are released and circulated in the body of "chronic habitual" and affect the other body parts. Like other food substances and drugs, the chemicals excreted from AN are metabolized in the liver and excreted through the kidney. They are thereby making these organs highly susceptible to the undesired consequences of AN, as seen in the oral cavity. In other words, the harmful effects of AN involve various body systems also. AN consumption may contribute to cardiac, endocrinal, and metabolic disorders. It may cause exacerbation of respiratory disorders also. AN has adverse effects on reproductive organs and their functions [5,21,29]. AN may cause liver fibrosis [30]. Thus, oral health professionals should have awareness of the ill effects of AN on various vital organs.

A review by Arora et al. [7] mentioned that dentists and dental auxiliaries should bring awareness to the general public regarding AN use. The dentists can identify the habitual though the patient avoids giving habit history as they are aware of the oral effects of AN use. A dentist is the right person to change the patient's behavior with reference to his AN habit. The studies proposed to have the policy to prohibit the manufacturing and sale of AN to curb usage [21]. According to another study, the role of psychological and pharmacotherapeutic interventions for AN cessation should be assessed [31]. 

It is reported that AN affect almost all organs in the human body. Chewing causes as well as worsens systemic disorders in humans, which include neurological, cardiac, endocrine, liver, and respiratory diseases. Chewing has an impact on central obesity and the reproductive system. Chronic AN consumption results in the suppression of immunity in humans [5].

Wu et al. [32] evaluated the probable effects of BQ chewing on mortality in a cohort study. They observed a positive relationship between the dose and duration of AN chewing and mortality. Although the adverse effects of AN consumption are extensively reported, there are certain favorable effects of AN mentioned in the literature.

Favorable Effects of AN on the Oral and General Health

Beneficial effects of AN on the oral cavity include teeth cleansing and strengthening of teeth, jaws, and tongue. It is believed that pan chewing with AN prevents caries. It prevents oral bad breadth also. The effect of AN on the gastrointestinal system includes the facilitation of bowel movements and the prevention of parasitic infections [3,20,33,34].

AN chewing increases attentiveness and energy. It causes euphoria due to its arecoline content as arecoline is an agonist of muscarinic acetylcholine receptors. There are a number of favorable effects of AN on the nervous system, which include improvement in concentration and relaxation, lifting mood, controlling hunger, aphrodisiac properties, and post-prandial digestant [33]. Arecoline of AN activates nicotine acetylcholine receptors, thus causing nicotine addiction. This is a reason for encouraging the addition of tobacco to AN. Areca husk exhibits antifungal properties through substrate deprivation and membrane disruption of the fungal organism. Tannins form a complex with iron ions that interfere with the Fenton reaction and exhibit their antioxidant activity [35]. Accordingly, Jaiswal et al. [36] reported AN as a valuable herbal medicine against several problems.

The important point to understand is, though there are few beneficial actions of AN, once an individual becomes a habitual AN chewer, the harmful effects outweigh the beneficial effects. The prevalence of consumption of AN is increasing day by day, and the population should be made aware of the health issues related to it, as the health of consumers is at stake.

Casual use of small quantities of AN with or without tobacco regularly results in dependency syndrome and then becomes a habit. The consumption of AN and its harmful effects on human health is not an issue associated with an individual or a few people, but it becomes a community problem. Necessary actions to control this issue are required at the individual level, community level, and national levels. A policy to prohibit the manufacturing and sale of AN to curb usage should exist.

## Conclusions

AN consumption habit is quite popular in India. Though there are few useful effects of AN, the harmful effects overweight the beneficial effects. AN habit often acts as a starter for tobacco habit. The present review describes that AN is capable of causing a number of harmful effects on human health.

Considering the complex composition of AN and its adverse effects on oral and general health, it is better to act late than never. In regard to controlling the issue about the harmful effects of this habit, preventive measures should be established. The process of regulating the detrimental impact of AN begins by exploring the habit history of all the subjects reporting to dental OPD and to do counseling for AN cessation.
